# Protective effect of etanercept, an inhibitor of tumor necrosis factor-α, in a rat model of retinal ischemia

**DOI:** 10.1186/s12886-016-0262-9

**Published:** 2016-06-04

**Authors:** Hyoung Won Bae, Naeun Lee, Gong Je Seong, Seungsoo Rho, Samin Hong, Chan Yun Kim

**Affiliations:** Department of Ophthalmology, Severance Hospital, Institute of Vision Research, Yonsei University College of Medicine, 134 Shinchon-dong, Seodaemun-gu, Seoul, South Korea; Department of Ophthalmology, Hallym Hospital, Incheon, South Korea; Department of Ophthalmology, CHA Bundang Medical Center, CHA University, Seongnam, South Korea

**Keywords:** Etanercept, Tumor necrosis factor-α, Acute ischemia, Axonal injury, Microglia

## Abstract

**Background:**

To assess the neuroprotective effect of etanercept (Enbrel®) which is a commercialized Tumor necrosis factor-α (TNF-α) inhibitor on axonal injury in an animal model of acute ischemia.

**Methods:**

Acute ischemia was induced by intraocular pressure elevation in 36 rats. The treatment groups underwent subcutaneous injection of etanercept (0.3 or 1.0 mg/kg) three times per week up to 4 weeks. The control groups were treated in the same manner using the same volume of phosphate-buffered saline (PBS). Optic nerve damage was evaluated by counting the number of axons under a transmission electron microscope. Microglial cell activity was assessed using Iba1 and CD68.

**Results:**

After induction of ischemia, the ratio of preserved axons was significantly greater in the 2-week 1.0-mg/kg etanercept-treated group than in the PBS-treated group (*p* = 0.062). The 4-week 0.3-mg/kg and 1.0-mg/kg etanercept-treated groups also showed significantly higher ratios of preserved axons than did the PBS-treated group (*p* = 0.021 and 0.003, respectively). The expression of Iba1 and CD68 in the optic nerve was lower in the etanercept-treated groups than in the PBS-treated groups. Immunohistochemical staining using rabbit anti-Iba1 antibody showed that the amount of microglia at the optic nerve head was noticeably lower in the etanercept-treated groups than in the PBS-treated groups.

**Conclusions:**

Etanercept significantly suppressed optic nerve injury in this rat model of acute ischemia. This in vivo study suggests that etanercept might be a novel neuroprotective treatment agent for TNF-α–related disease.

## Background

Ischemia is considered to be a major pathogenic condition underlying several eye diseases including diabetic retinopathy, retinal arterial/vein occlusion, and glaucoma [1, 2]. Ischemia leads to apoptotic cell death through activation of glial cells, increasing the production of certain neurotoxic mediators [3, 4].

Tumor necrosis factor-α (TNF-α), an inflammatory cytokine, is one such factor that increases after ischemic injury; it is also secreted in response to inflammation, infection, and trauma [5]. TNF-α induces apoptotic neuronal damage through various signaling events within cells after binding to its receptor [6]. Previous studies have shown increased TNF-α production in in vitro and in vivo ischemia models [3, 7, 8] and have demonstrated that neutralization of TNF-α by intravitreal TNF-α antibody injection significantly preserves retinal function [8].

Etanercept (Enbrel®) is a TNF-α inhibitor that functions as a decoy receptor that binds to TNF-α. It was the first fusional monoclonal antibody against TNF-α to be marketed for clinical use and is now commercially available. Etanercept reduces the inflammatory effect of TNF-α and is used clinically to treat several autoimmune diseases such as rheumatoid arthritis, ankylosing spondylitis, and psoriatic arthritis [9, 10]. Additionally, a recent study showed that systemically injected etanercept effectively prevented retinal ganglion cell loss in a rat model of glaucoma in which TNF-α and its receptor were detected at high levels, as well as in rats with endotoxin-induced uveitis [11].

In the present study, we administered etanercept, a widely used TNF-α inhibitor, subcutaneously after acute ischemic injury to ascertain its effectiveness in preventing axonal ischemic damage. The aim of this study was to determine whether etanercept reduces optic nerve degeneration and decreases microglia activation in a rat model of high-pressure-induced acute retinal ischemia.

## Methods

### Animal and retinal ischemia

Thirty-six male Sprague–Dawley rats weighing 250 to 300 g were used in this study. All animals were housed in a standard animal facility with *ad libitum* access to food and water in a room with a 12-h light–dark cycle at constant temperature of 21 °C. All experimental procedures conformed to the Association for Research in Vision and Ophthalmology Statement for the Use of Animals in Ophthalmic and Vision Research. The animal protocols were approved by the Institutional Animal Care and Use Committee of Yonsei University Medical Center.

Before induction of ischemia, the rats were placed under anesthesia by an intraperitoneal injection of 30 mg/kg of tiletamine + zolazepam (Zoletil; Virbac, Fort Worth, TX) and 10 mg/kg of xylazine (Rompun 2 %; Bayer, Peoria, IL). Ischemia was induced by increasing the intraocular pressure (IOP), thus blocking the blood supply from the retinal artery to the retina. The anterior chamber of the right eye was cannulated with a 30-gauge needle attached to silastic tubing and a manometer to allow for infusion of sterile 0.9 % saline solution. The IOP was increased by raising the saline container to exceed the systemic arterial blood pressure. An IOP of 130 mmHg was maintained for 60 min [8]. Whitening of the iris and loss of the red reflex of the retina confirmed retinal ischemia. The IOP was monitored every 5 min, and the absence of retinal perfusion was maintained. The infusion was then stopped to allow for reperfusion of the retinal vasculature, which was confirmed by reappearance of the red reflex. The contralateral left eye was treated by insertion of a 30-gauge needle into the anterior chamber through the cornea without infusion, thus serving as a nonischemic control. The animals were humanely killed at various time points, and their eyes were enucleated for morphologic and immunohistochemical studies.

### Treatment with etanercept

We reconstituted etanercept (Enbrel®; Amgen, Thousand Oaks, CA) with sterile water to 0.3 or 1.0 mg/kg. The rats were distributed into three groups. Starting 1 day after induction of acute ischemia or sham injection, the first and second groups underwent subcutaneous injections of etanercept at 0.3 mg/kg (n = 6) and 1.0 mg/kg (n = 15), respectively, in the scalp three times per week until the day of sacrifice. The second group was treated with 1.0 mg/kg etanercept, and three animals were killed after 3 days, six after 2 weeks, and six after 4 weeks. The third group (n = 15) was treated in the same manner with the same volume of phosphate-buffered saline (PBS); three animals were killed after 3 days, six after 2 weeks, and six after 4 weeks. These doses were selected based upon previous studies that demonstrated the effectiveness of the drug in other disease models [11].

### Histological evaluation and immunohistochemistry

Initially, the eyeball was enucleated under anesthesia. To minimize stretching damage during the enucleation procedure, the orbital part of the optic nerve was dissected through a lateral conjunctival incision with a lateral canthotomy. When the perineurium was visualized enough to obtain an appropriate nerve length for the embedding procedure, the optic nerve was cut approximately 3 mm from the stump and removed from the eyeball. The acquired axons were fixed in Karnovsky’s solution and osmicated with 1 % osmium tetroxide, then processed for routine paraffin embedding. The globes were sagittally embedded, and 10-μm serial sections were cut in all cases. The short piece of the proximal optic nerve was taken for histology and fixed by immersion in 2.5 % glutaraldehyde with 4 % paraformaldehyde in 0.1 M phosphate buffer (pH 7.4) for 24 h at 4 °C. It was then placed in 1 % osmium tetroxide in saline overnight and washed with cacodylate buffer at room temperature. The tissue was subsequently dehydrated in a graded alcohol series and embedded in epoxy resin (Ladd Research Industries, Burlington, VT). Semithin (<1.0-μm) cross sections of the optic nerve (obtained from the midpoint of the sample, approximately 1.5 mm from the stump) were stained with 1 % toluidine blue in 1 % sodium borate to measure the cross-sectional area. Ultrathin (60-nm) cross sections were prepared for transmission electron microscopy (TEM) (EM410; Philips, Eindhoven, Netherlands).

The optic nerve, which was obtained 3 days after induction of ischemic injury, was used for immunohistochemical assessment of microglial activity. Retinal sections (10 mm) with the optic nerve attached were preblocked (PBS containing 10 % goat serum, 0.5 % gelatin, 3 % BSA, and 0.2 % Tween-20) and then incubated with rabbit anti-Iba1 antibody (1:500; Wako Chemicals USA Inc., Richmond, VA) as a microglial marker.

### Quantification of optic nerve axon loss

The present study was based on the premise that the cross sections of optic nerve axons are circular or oval in shape and pertinent in size. Degenerated axons must lose their circularity and deviate from the normal size range. Axonal degeneration is characterized by swollen axons and splitting of the myelin sheath into layers (hyperdense axons) with myelin sheath disruption and extensive fibrosis in cases of severe damage.

Our methodology for quantifying the damage to the optic nerve axon is described in detail elsewhere [12]. Briefly, 10 standard rectangular regions were randomly selected from each section and photographed at 3000× magnification. Nonaxonal regions were edited from each photograph using ImageJ (developed by Wayne Rasband, National Institutes of Health, Bethesda, MD; available at http://rsb.info.nih.gov/ij/index.html) and Windows Paint (Windows 7; Microsoft, Redmond, WA). Averages were calculated by counting the number and averaged areas of axoplasm (size of each axon internal to its myelin sheath [13]) using the particle analysis plug-in within the ImageJ software [14]. We assessed the axon damage with the above averaged number and area of axoplasm, and the sum of the calculated area was used to estimate the actual area by comparing the proportion and size of each TEM slide (1200 μm^2^).

### Western blot analysis

Three days after induction of acute ischemic injury, the microglial cell markers Iba1 (1:250; Abcam) and CD68 (1:100; Santa Cruz) were subjected to western blot analysis. Equal amounts of protein were electrophoresed in 10 % SDS-polyacrylamide gels. Separated proteins were then electrotransferred to PVDF membranes (0.45 μm; Millipore, Bedford, MA). After blocking with 5 % skim milk, the membranes were incubated overnight with a primary antibody against Iba1 and CD68. The blotted membranes were then incubated for 1 h at room temperature with an HRP-conjugated secondary antibody directed against rabbit IgG (1:2,000; Cell Signaling). Immunoreactive bands were visualized by enhanced chemiluminescence and exposed onto X-ray film (AGFA, Belgium).

### Statistical analysis

Results are expressed as means ± standard error of the mean. Statistical analysis was performed nonparametrically using the Mann–Whitney *U*-test (SPSS Statistics 19.0; Chicago, IL). A *p*-value of <0.05 was considered to indicate statistical significance.

## Results

### Etanercept reduced pathologic changes in axons

**Fig. 1:**
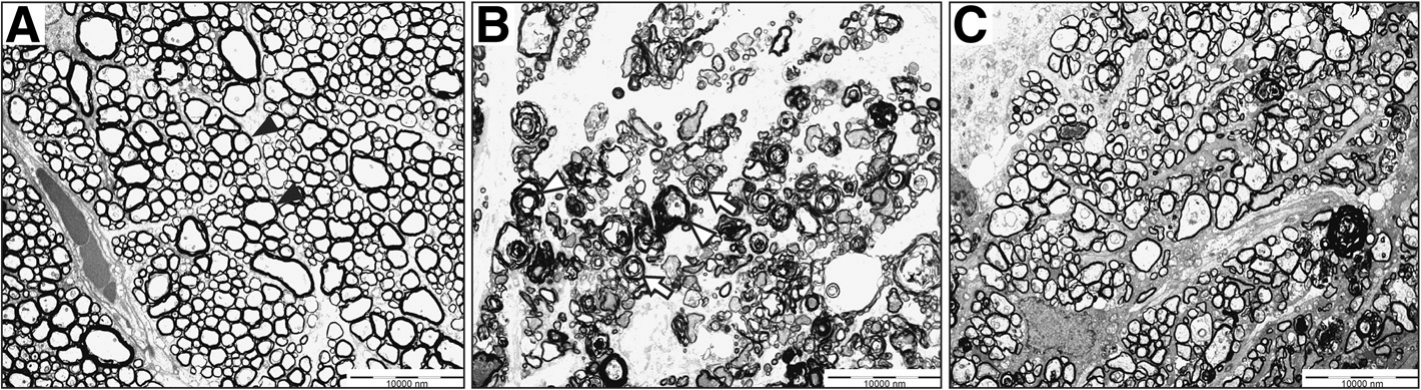
Etanercept prevents axonal damage secondary to ischemic injury. **a**-**c** Transmission electron micrographs of cross sections through rat optic nerves. **a** Normal compact axons with myelination and round shape in sham-treated control optic nerves (black arrowhead). **b** Axonal shrinkage with distortion of round shape and demyelination 4 weeks after acute ischemic injury. Separation of myelin sheath (white arrowhead) and a whorl-shaped mass (white arrow). **c** Relatively preserved round axons with myelination in ischemic model treated with 1.0 mg/kg etanercept. Original magnification: ×400; Scale bar, 10 μm

We compared the axon morphology and density in cross sections of the optic nerve under TEM. Four weeks after induction of acute ischemia, the optic nerve showed markedly greater axonal disorder than did that of sham-treated controls; this disorder was characterized by axonal swelling with separation of the myelin sheath, increased variability in axonal size and shape, and decreased axonal density (Fig. 1a and b). However, the axons in eyes treated with 1.0 mg/kg of etanercept for 4 weeks maintained a more normal configuration and a density similar to that of the control axons (Fig. 1c).

The ratio of preserved axons was 0.88 ± 0.16 and 0.68 ± 0.17 in the 2-week 1.0 mg/kg etanercept-treated group and PBS-treated group, respectively, with a marginally significant difference (*p* = 0.062) (Table 1). The ratio of preserved axons was 0.98 ± 0.04 and 0.65 ± 0.11 in the 4-week 1.0-mg/kg etanercept-treated group and PBS-treated group, respectively, with a statistically significant difference (*p* = 0.003). The 4-week 0.3 mg/kg etanercept-treated group also demonstrated significantly greater axonal conservation than did the PBS-treated group (*p* = 0.021).

**Table 1:** Comparison of the ratio of preserved axons in the etanercept and control groups

Etanercept treatment group		PBS treatment group	*p*-value
1.0 mg/kg for 2 weeks	0.88 ± 0.16	0.68 ± 0.17	0.062
1.0 mg/kg for 4 weeks	0.98 ± 0.04	0.65 ± 0.11	0.003
0.3 mg/kg for 4 weeks	0.78 ± 0.40	0.65 ± 0.08	0.021

*PBS* phosphate-buffered saline

### Etanercept decreased microglial activation

**Fig. 2:**
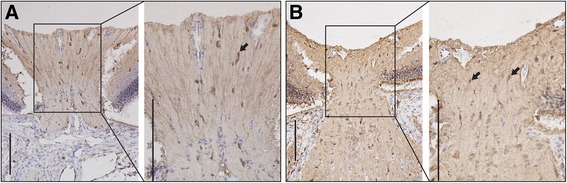
Immunohistochemical staining 3 days after acute ischemic injury. Using a rabbit anti-Iba1 antibody, immunohistochemical staining showed that the number of microglia (arrow) at the optic nerve head was noticeably lower in the 1.0 mg/kg etanercept-treated group (**a**) than in the PBS-treated group (**b**). Original magnification: ×20 (A, B); Scale bar, 1000 μm. PBS, phosphate-buffered saline

**Fig. 3:**
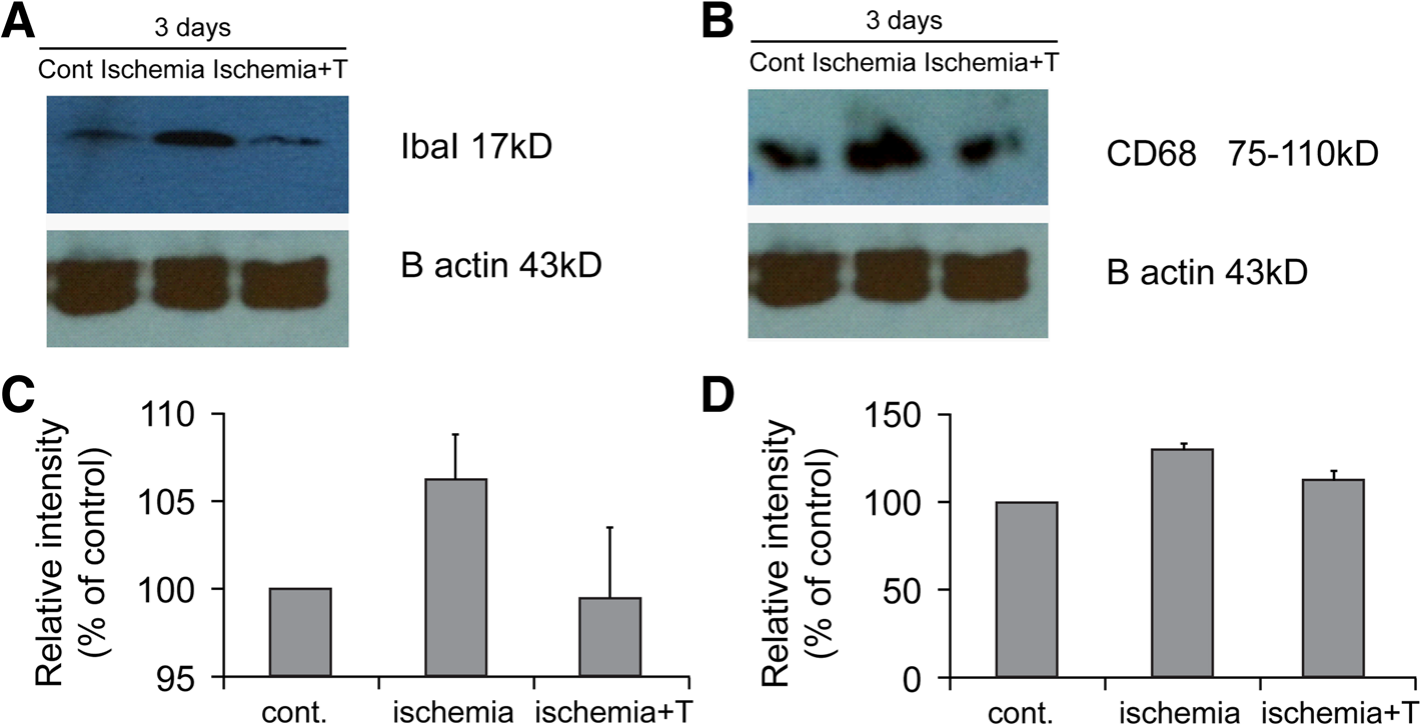
Western blot analysis of Iba1 and CD68 3 days after establishment of acute ischemia. Differences in loading were normalized to the level of beta-actin. **a**, **b** Low expression of both Iba1 and CD68 is seen in the control and 1.0 mg/kg etanercept-treated ischemic groups, while distinct protein bands corresponding to Iba1 and CD68 are seen in the untreated ischemic group. **c**, **d** Levels of Iba1 and CD68 relative to beta-actin. **a**, **c** Iba1. **b**, **d** CD68. Cont, sham-treated control model; Ischemia, untreated ischemic group; Ischemia + T, ischemia with 1.0 mg/kg etanercept treatment group; B actin, beta-actin

Immunohistochemical staining using a rabbit anti-Iba1 antibody showed that the amount of microglia at the optic nerve head was noticeably lower in the 1.0 mg/kg etanercept-treated group than in the PBS-treated group on the third day after induction of acute ischemia (Fig. 2). The western blot Iba1 and CD68 expression results are shown in Fig. 3. In the control eyes without ischemic injury, few Iba1- and CD68-positive cells were present in the optic nerve. In the eyes with ischemia, on the other hand, distinct protein bands corresponding to Iba1 and CD68 were found with molecular weights of 17 kDa and 75–110 kDa, respectively. However, expression levels of both markers were markedly lower in the 1.0 mg/kg etanercept-treated eyes than in the control eyes. Therefore, treatment with etanercept appeared to have prevented the dramatic increase in Iba1 and CD68 expression following ischemic insult.

## Discussion

Etanercept is a commercially available TNF-α inhibitor, and the present study confirmed its efficacy in terms of preserving axonal structure and decreasing microglial activation in a rat model of retinal ischemia. Previous studies demonstrated that etanercept decreased intraocular inflammation in a rat model of uveitis and prevented retinal ganglion cell loss in a rat model of glaucoma by reducing the TNF-α level [11, 15]. To our knowledge, this is the first study to show the protective effect of etanercept against acute retinal ischemia in an in vivo model.

TNF-α plays an important role in ischemic neuronal damage, and elevated levels of TNF-α have been documented in several previous studies [3, 7, 8, 11]. Therefore, etanercept might also be effective for the prevention of axonal ischemic damage after acute ischemic injury. Actually, Berger et al [8]. demonstrated that intravitreal TNF-α antibody administration prevented ischemic retinal injury in a rat model. However, that study had drawbacks in that intravitreal injection could induce additional inflammation and increase the IOP. To overcome such limitations and assess the effect of lowering TNF-α, we used subcutaneous injections of etanercept. As a result, we confirmed that etanercept effectively prevents axonal ischemic damage after systemic administration.

Previous studies have also shown that microglia are the source of TNF-α and that the activation of these cells may quantitatively reflect the severity of optic nerve damage [3, 11, 16, 17]. Ischemic injury may break down the blood–retina barrier or cause local stress at the optic nerve head, subsequently eliciting a glial response. When microglia are activated, they rapidly transform by increasing the size of their cell body, thickening their processes, and decreasing the ramification of their distal branches until they become almost completely round. Functionally, activated microglia may continuously present antigens and generate several cytokines such as TNF-α, which plays an important role in the inflammatory response [18]. This microglial activation occurs along the entire optic pathway and seems to be closely correlated with axonal damage. Therefore, microglial activation could be a useful quantitative tool with which to assess the status of optic nerve damage in models of optic nerve injury.

In the present study, we confirmed the occurrence of microglial activation using microglial markers on the third day after induction of acute ischemia. Roh et al [11]. reported that the degree of elevation of the TNF-α level within 3 days after IOP elevation in a rat model of glaucoma was correlated with microglial activation by 7 days. Therefore, we evaluated microglial activation 3 days after ischemic injury to elucidate the microglial-induced inflammatory reaction. Two microglial markers, Iba1 and CD68, were used in this study. Iba1 labels both quiescent and activated microglia and provided an index of microglial density, whereas lysosomal antigen CD68 served as a measure of microglial phagocytic activity [19].

We used a semi-automated counting method by TEM in which we calculated the ratio of preserved axons to assess the axonal damage [20]. Marina et al [14]. described a semi-automated targeted sampling method using light micrographs, and we applied this technique to our TEM slides. By minimizing the proportion of subjective procedures and using TEM photographs, we quantitatively evaluated the status of the axons with less labor and avoided the possibility of underestimation, which might occur using light microscopy. Using this method, we found that the number and configuration of axons after acute ischemic injury were significantly more preserved in the etanercept-treated group than in the PBS-treated group.

This study had several limitations. First, we did not directly evaluate retinal ganglion cell loss or the TNF-α level. However, retinal ganglion cell loss after ischemia has been described in many previous studies, and the TNF-α–lowering effect of etanercept is also apparent. This study aimed to demonstrate the actual effects of commercialized etanercept on acute ischemic injury. Therefore, after induction of acute ischemia, we determined whether etanercept suppresses microglial activity and thus lowers the TNF-α level, helping to preserve the axons of the optic nerve. Second, axonal damage might be induced by both mechanical and ischemic damage. A very high IOP was required to achieve an ischemic condition. During this process, the optic nerve head might have sustained mechanical damage caused by the elevated pressure. Therefore, we cannot conclusively assert that the axons were damaged only by the ischemic condition. Finally, we could not determine the optimal dose and duration of etanercept. Although the effect of etanercept showed a tendency to increase with the dosage and duration, further studies to investigate the clinical use of etanercept are warranted.

Even considering these limitations, this study has strength of the first study which showed the protective effect of commercialized etanercept against acute retinal ischemia in an in vivo model.

## Conclusions

Etanercept, a commercially available TNF-α inhibitor, showed a significant protective effect against axonal damage after induction of acute ischemia in in vivo rat models. Additional studies on the neuroprotective effect of etanercept will help to establish whether this agent is a good candidate for novel treatment of various neuronal diseases.

## Abbreviations

IOP, intraocular pressure; PBS, phosphate-buffered saline; TEM, transmission electron microscopy; TNF-α, tumor necrosis factor-α

## References

[1] Quigley HA, Addicks EM (1980). Chronic experimental glaucoma in primates. II. Effect of extended intraocular pressure elevation on optic nerve head and axonal transport. Invest Ophthalmol Vis Sci.

[2] Osborne NN, Casson RJ, Wood JP, Chidlow G, Graham M, Melena J (2004). Retinal ischemia: mechanisms of damage and potential therapeutic strategies. Prog Retin Eye Res.

[3] Tezel G, Wax MB (2000). Increased production of tumor necrosis factor-alpha by glial cells exposed to simulated ischemia or elevated hydrostatic pressure induces apoptosis in cocultured retinal ganglion cells. J Neurosci.

[4] Buchi ER (1992). Cell death in the rat retina after a pressure-induced ischaemia-reperfusion insult: an electron microscopic study. I. Ganglion cell layer and inner nuclear layer. Exp Eye Res.

[5] Smith CA, Farrah T, Goodwin RG (1994). The TNF receptor superfamily of cellular and viral proteins: activation, costimulation, and death. Cell.

[6] Wajant H, Pfizenmaier K, Scheurich P (2003). Tumor necrosis factor signaling. Cell Death Differ.

[7] Gesslein B, Hakansson G, Gustafsson L, Ekstrom P, Malmsjo M (2010). Tumor necrosis factor and its receptors in the neuroretina and retinal vasculature after ischemia-reperfusion injury in the pig retina. Mol Vis.

[8] Berger S, Savitz SI, Nijhawan S, Singh M, David J, Rosenbaum PS, Rosenbaum DM (2008). Deleterious role of TNF-alpha in retinal ischemia-reperfusion injury. Invest Ophthalmol Vis Sci.

[9] Nanda S, Bathon JM (2004). Etanercept: a clinical review of current and emerging indications. Expert Opin Pharmacother.

[10] Ramiro S, Radner H, van der Heijde D, van Tubergen A, Buchbinder R, Aletaha D, Landewe RB. Combination therapy for pain management in inflammatory arthritis (rheumatoid arthritis, ankylosing spondylitis, psoriatic arthritis, other spondyloarthritis). Cochrane Database Syst Rev. 2011(10):Cd008886. 10.1002/14651858.CD008886.pub2 PMC12416524 21975788

[11] Roh M, Zhang Y, Murakami Y, Thanos A, Lee SC, Vavvas DG, Benowitz LI, Miller JW (2012). Etanercept, a widely used inhibitor of tumor necrosis factor-alpha (TNF-alpha), prevents retinal ganglion cell loss in a rat model of glaucoma. PLoS One.

[12] Rho S, Park I, Seong GJ, Lee N, Lee CK, Hong S, Kim CY (2014). Chronic ocular hypertensive rat model using microbead injection: comparison of polyurethane, polymethylmethacrylate, silica and polystyene microbeads. Curr Eye Res.

[13] DeMaman AS, Melo P, Homem JM, Tavares MA, Lachat JJ (2010). Effectiveness of iron repletion in the diet for the optic nerve development of anaemic rats. Eye (Lond).

[14] Marina N, Bull ND, Martin KR (2010). A semiautomated targeted sampling method to assess optic nerve axonal loss in a rat model of glaucoma. Nat Protoc.

[15] Avunduk MC, Avunduk AM, Oztekin E, Baltaci AK, Ozyazgan Y, Mogolkoc R (2004). Etanercept treatment in the endotoxin-induced uveitis of rats. Exp Eye Res.

[16] Ebneter A, Casson RJ, Wood JP, Chidlow G (2010). Microglial activation in the visual pathway in experimental glaucoma: spatiotemporal characterization and correlation with axonal injury. Invest Ophthalmol Vis Sci.

[17] Yuan L, Neufeld AH (2001). Activated microglia in the human glaucomatous optic nerve head. J Neurosci Res.

[18] Schwaiger FW, Hager G, Raivich G, Kreutzberg GW (1998). Cellular activation in neuroregeneration. Prog Brain Res.

[19] Nimmerjahn A, Kirchhoff F, Helmchen F (2005). Resting microglial cells are highly dynamic surveillants of brain parenchyma in vivo. Science.

[20] Kim CY, Rho S, Lee N, Lee CK, Sung Y. Semi-automated counting method of axons in transmission electron microscopic images. Vet Ophthalmol. 2016;19(1):29-37. 10.1111/vop.12247 25639186

